# Acute kidney injury due to thin basement membrane disease mimicking Deferasirox nephrotoxicity: a case report

**DOI:** 10.1186/s12882-018-1180-2

**Published:** 2018-12-17

**Authors:** Keiko Oda, Kan Katayama, Akiko Tanoue, Tomohiro Murata, Yumi Hirota, Shoko Mizoguchi, Yosuke Hirabayashi, Takayasu Ito, Eiji Ishikawa, Kaoru Dohi, Masaaki Ito

**Affiliations:** Department of Cardiology and Nephrology, Mie University Graduate School of Medicine, 2-174 Edobashi, Tsu, Mie 514-8507 Japan

**Keywords:** Acute kidney injury, Deferasirox - gross hematuria - myelodysplastic syndrome - thin basement membrane disease

## Abstract

**Background:**

Although the renal toxicity of Deferasirox, an oral iron chelator, has been reported to be mild, there have been reports of acute interstitial nephritis or Fanconi syndrome due to this agent. Thin basement membrane disease (TBMD) is a hereditary disease characterized primarily by hematuria, with gross hematuria also observed in about 7% of cases. We herein report a case of TBMD that presented with acute kidney injury and gross hematuria during treatment with Deferasirox.

**Case presentation:**

The patient was a 63-year-old man who had been diagnosed with myelodysplastic syndrome 6 years ago. He had started taking Deferasirox at 125 mg due to post-transfusion iron overload 6 months ago. Deferasirox was then increased to 1000 mg three months ago. When the serum creatinine level increased, Deferasirox was reduced to 500 mg three weeks before hospitalization. Although the serum creatinine level decreased once, he developed a fever and macroscopic hematuria one week before hospitalization. The serum creatinine level increased again, and Deferasirox was stopped four days before hospitalization. He was admitted for the evaluation of acute kidney injury and gross hematuria. Treatment with temporary hemodialysis was required, and a kidney biopsy was performed on the eighth day of admission. Although there was no major abnormality in the glomeruli, the leakage of red blood cells into the Bowman’s space was observed. Erythrocyte cast formation was observed in the tubular lumen, which was associated with acute tubular necrosis. The results of an electron microscopic study were compatible with TBMD.

**Conclusion:**

Although Deferasirox is known to be nephrotoxic, gross hematuria is relatively rare. When we encounter a case of acute kidney injury with gross hematuria during treatment with Deferasirox, TBMD should be considered as a possible cause of gross hematuria.

## Background

Deferasirox is an oral iron chelator used for treating chronic iron overload in transfused hematologic patients, such as those with thalassemia major and myelodysplastic syndrome (MDS). Although the renal toxicity of Deferasirox is reportedly mild [1], there have been reports of acute interstitial nephritis or Fanconi syndrome due to this agent [2–11].

Thin basement membrane disease (TBMD) is a hereditary disease thought to be caused by heterozygous mutations in COL4A3 or COL4A4. Although microscopic hematuria is characteristic of TBMD and the clinical course of TBMD is good, gross hematuria is also observed in about 7% of cases [12].

We herein report a case of TBMD that presented with acute kidney injury and gross hematuria during treatment with Deferasirox.

## Case presentation

The patient was a 63-year-old man who had neither microscopic nor macroscopic hematuria at his previous medical checkup. There was no apparent family history of kidney disease. His leukocyte and platelet counts had begun to decrease 6 years ago, and he was diagnosed with myelodysplastic syndrome (MDS) by bone marrow aspiration. He started treatment for anemia with blood transfusion 2 years ago. He was not on any antiplatelet or anticoagulant medications and his serum creatinine (Cr) level was 0.74 mg/dL 9 months previously. He started taking the oral iron chelator Deferasirox at 125 mg due to post-transfusion iron overload 6 months ago. The dosage was then increased to 1000 mg 3 months ago. When the serum Cr level increased, the Deferasirox dosage was reduced to 500 mg 3 weeks before hospitalization. Although the serum Cr level decreased once, he developed a fever and macroscopic hematuria 1 week before hospitalization. The serum Cr level increased again, and Deferasirox was stopped 4 days before hospitalization. He was admitted urgently to our hospital for the evaluation of acute kidney injury and macroscopic hematuria.

On admission, his body temperature was 36.7 °C, and his heart rate was 81 per minute. His blood pressure was 125/64 mmHg. A physical examination revealed no abnormalities except for anemic palpebral conjunctiva. The laboratory values at the time of hospitalization are shown in Table 1. Pancytopenia was noted, and the coagulation values were within the respective normal ranges. The blood urea nitrogen and serum Cr levels were 42.0 and 3.97 mg/dL, respectively. The serum ferritin level was high at 14230 ng/mL. A urinalysis showed proteinuria and hematuria, and a urinary sediment analysis revealed more than 100 dysmorphic red blood cells (RBCs) per high-power field with epithelial casts, granular casts, and RBC casts. The urinary protein-to-creatinine ratio was 1.20 g/gCr. Urinary N-acetyl-β-D-glucosaminidase and β_2_ microglobulin values were 18.4 g/gCr and 2757 μg/L, respectively. The myeloperoxidase-antineutrophil cytoplasmic antibody (MPO-ANCA), proteinase-3-ANCA, anti-glomerular basement membrane (GBM) antibody, antinuclear antibody, and anti-streptolysin O were all negative.

**Table 1:** The laboratory data on admission

Parameter	Patient value	Reference
Urinalysis		
pH	5.5	4.5–7.5
Protein (g/gCr)	1.2	< 0.15
Occult blood	3+	(−)
Dysmorphic red blood cells (/HPF)	> 100	< 5
Epithelial casts (/WF)	20–29	(−)
Granular casts (/WF)	20–29	(−)
Red blood cell casts (/WF)	1–4	(−)
N-acetyl--D-glucosaminidase (U/gCr)	18.4	< 5.6
β2microglobulin (*μ*g/L)	2757	< 150
Hematology		
White blood cell count (/μL)	2070	3300–8600
Neutrophils (%)	59.0	48.0–61.0
Red blood cells (× 10^4^/μL)	240	435–555
Hemoglobin (g/dL)	7.4	13.7–16.8
Hematocrit (%)	22.8	40.7–50.1
Platelet count (× 10^4^/*μ*L)	11.4	15.8–34.8
Coagulation		
Activated partial thromboplastin time (seconds)	27.7	< 37.0
Prothrombin time (seconds)	12.8	9.8–12.1
Prothrombin time international normalized ratio	1.11	0.88–1.08
Fibrinogen (mg/dL)	660	200–400
Blood chemistry		
Total protein (g/dL)	6.5	6.6–8.1
Albumin (g/dL)	3.6	4.1–5.1
Blood urea nitrogen (mg/dL)	42.0	8.0–20.0
Creatinine (mg/dL)	3.97	0.65–1.07
Lactic dehydrogenation enzyme (U/L)	384	124–222
Uric acid (mg/dL)	8.9	3.7–7.8
Natrium (mmol/L)	137	138–145
Potassium (mmol/L)	5.1	3.6–4.8
Chlorine (mmo/L)	101	101–108
Calcium (mg/dL)	9.2	8.8–10.1
Inorganic phosphates (mg/dL)	3.9	2.7–4.6
Bicarbonate ion (mmol/L)	22	22–32
C-reactive protein (mg/dL)	5.12	< 0.14
Fe (*μ*g/dL)	171	40–188
Total iron-binding capacity (μg/dL)	221	253–365
Ferritin (ng/mL)	14,230	50.0–200.0

After hospitalization, the patient was treated with intravenous administration of 500 mg of methylprednisolone for 3 consecutive days, followed by 30 mg of prednisolone per day orally. Hemodialysis therapy was initiated due to oliguria and discontinued after 10 days because his urine volume recovered. A kidney biopsy was performed on the eighth day of admission.

**Fig. 1:**
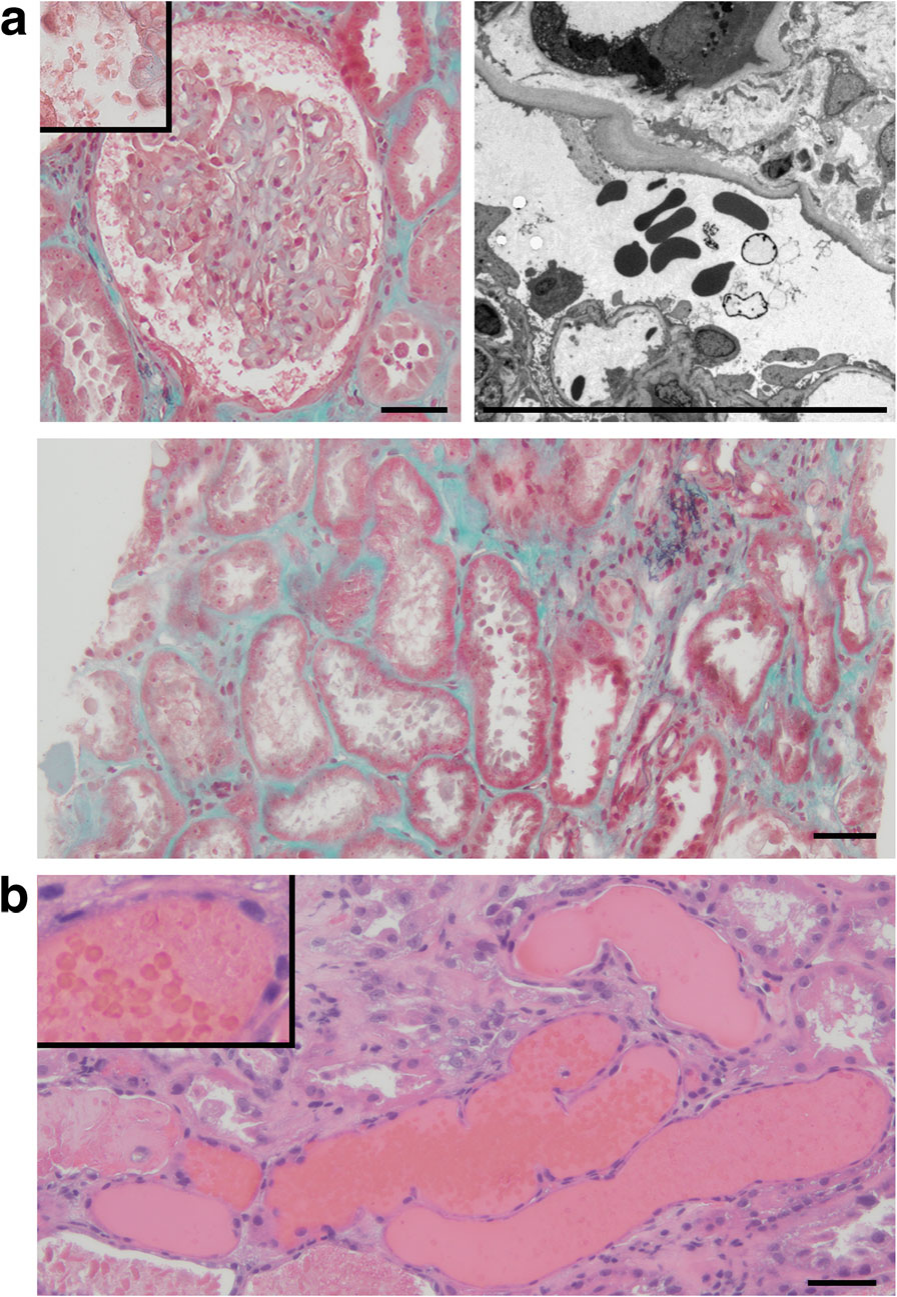
**a** Although there were no major abnormalities in the glomeruli (left-upper panel), the leakage of red blood cells into the Bowman’s space was observed (Masson Trichrome staining; the inset of left-upper panel). An electron micrograph showed the leakage of red blood cells, two of which were dysmorphic, into Bowman’s space (right-upper panel). Tubulointerstitial fibrosis with acute tubular necrosis was detected (Masson Trichrome staining; lower panel). **b** Massive erythrocyte cast formation was observed in the tubular lumen and the inset denoted red blood cells in Hematoxylin and eosin staining. Scale bars, 50 μm

**Fig. 2:**
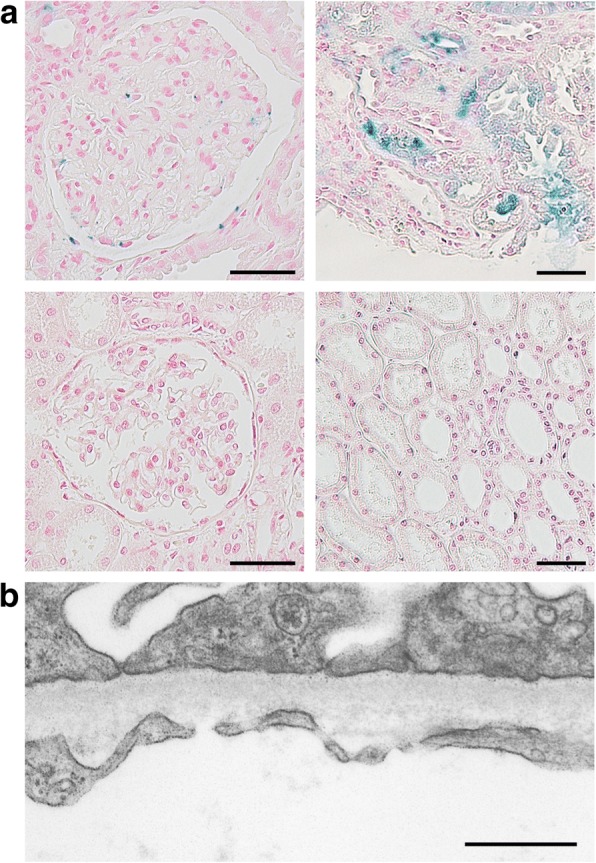
**a** There were positive blue signals in both the glomerular cells and Bowman’s capsular epithelial cells in Berlin blue staining (left-upper panel). The positive blue signals were prominent in the tubular cells (right-upper panel). There were no apparent positive blue signals in the normal human kidney sections (lower panels). Scale bars, 50 μm. **b** The results of an electron microscopic study revealed the thinning of the glomerular basement membrane without any immune complexes. Scale bar, 500 nm

One of 11 glomeruli was globally sclerotic. None of glomeruli showed crescentic formation or adhesion. Although there were no major abnormalities in the glomeruli (left-upper panel, Fig. 1a), the leakage of RBCs into the Bowman’s space was observed (inset of left-upper panel, Fig. 1a). An electron micrograph showed leakage of red blood cells, two of which were dysmorphic, into Bowman’s space (right-upper panel, Fig. 1a). Interstitial fibrosis with acute tubular necrosis were observed in approximately 40% of the total area (lower panel, Fig. 1a). Erythrocyte cast formation was observed in the tubular lumen, which was associated with tubular simplification (Fig. 1b). There were positive blue signals both in glomerular cells and Bowman’s capsular epithelial cells on Berlin blue staining, which was reflected glomerular hemorrhaging (left-upper panel, Fig. 2a). The positive blue signals were prominent in the tubular cells (right-upper panel, Fig. 2a). There were no apparent positive blue signals in the normal human kidney sections, which were purchased from Zyagen (HP-901, CA, USA) (lower panels, Fig. 2a). There was no significant staining for immunoglobulin G (IgG), IgA, IgM, complement 3 (C3), C1q, or fibrinogen.

Since there were no signs of rapidly progressive glomerulonephritis on a kidney biopsy, the amount of prednisolone after the second round of the intravenous administration of 500 mg of methylprednisolone for 3 consecutive days was decreased. Although the macroscopic hematuria disappeared at 2 weeks after admission, the serum Cr level was 1.92 mg/dL at the time of discharge and was unchanged after 2 months. The results of the electron microscopic study revealed thinning of the GBM without any immune complexes (Fig. 2b). The thickness of the average GBM was 199 ± 47 nm, which was consistent with TBMD. Therefore, a genomic sequence analysis of the COL4A3 and COL4A4 genes was performed by Sanger sequencing method after purifying the polymerase chain reaction products of exons, and five and six missense mutations were found in COL4A3 and COL4A4, respectively (Table 2).

**Table 2:** The results of the genomic sequence analysis

COL4A3			
c.127G > C	p.G43R	heterozygous	benign
c.422 T > C	p.L141P	homozygous	benign
c.485A > G	p.E162G	homozygous	benign
c.805G > A	p.E269K	heterozygous	benign/likely benign
c.1721C > T	p.P574L	heterozygous	benign
COL4A4			
c.839 T > G	p.M280R	heterozygous	unreported
c.1444C > T	p.P482S	heterozygous	benign
c.2656C > A	p.L886I	heterozygous	synonymous
c.3011C > T	p.P1004L	heterozygous	benign
c.3979G > A	p.V1327 M	homozygous	benign
c.4207 T > C	p.S1403P	homozygous	benign

## Discussion and conclusion

We encountered a patient who presented with acute kidney injury and gross hematuria during treatment with Deferasirox, which was used for transfusion-related iron overload due to MDS. A kidney biopsy revealed glomerular hemorrhaging and erythrocyte cast formation in the tubular lumen, which was associated with tubular simplification and acute tubular necrosis. An electron microscopic study revealed thinning of the GBM without any immune complexes, so the patient was diagnosed with TBMD.

Renal injury due to Deferasirox includes acute tubulointerstitial nephritis as well as Fanconi syndrome [2–11]. However, there has only been one report of mild microscopic hematuria [2], and gross hematuria as a side effect of Deferasirox is considered unlikely [13]. The present case showed progressive kidney injury despite the cessation of Deferasirox, and the presence of gross hematuria with dysmorphic RBCs and RBC casts in the urine sediment could not be explained by toxicity due to Deferasirox alone, since there was no evidence of acute tubulointerstitial nephritis in the renal interstitium.

Since the serum ferritin level was high and there was deposition of hemosiderin in both podocytes and Bowman’s epithelial cells in the present case, we considered the possibility of iron overload nephropathy. However, gross hematuria is reportedly rare, although proteinuria has been observed in an iron-overload rat model [14].

There were many RBC casts in the tubules, and the leakage of RBC cells into the Bowman’s space was observed. Therefore, we considered that the RBC casts might have caused acute tubular necrosis and subsequent acute kidney injury. Chronic interstitial fibrosis was thought to be due to age-related change, since the patient was neither hypertensive nor diabetic. The deposition of hemosiderin in both podocytes and Bowman’s epithelial cells was thought to be the result of glomerular hemorrhaging. There has been a report of acute kidney injury with gross hematuria [15], including IgA and warfarin-related nephropathy. The injury was thought to have been caused by not only the increased intratubular pressure due to the obstruction of RBC casts, but also due to the influence of heme-induced oxidative damage [15].

The GBM of the present case was less than 250 nm, so we diagnosed the patient with TBMD. Furthermore, a genetic analysis of the COL4A3 and COL4A4 genes revealed five and six missense mutations, respectively. Two mutations, c.839 T > G (p.M280R) and c.2656C > A (p.L886I) in COL4A4, were unreported, while nine mutations were reported to be benign in the Leiden Open Variation Database or ClinVar (Table 2). Although c.2656C > A (p.L886I) in COL4A4 seemed to be a synonymous mutation, c.839 T > G (p.M280R) in COL4A4 has never been reported (submission code #00206809 in LOVD mutation database) and may be related to the onset of TBMD. However, c.839 T > G (p.M280R) in COL4A4 was predicted to be “benign” in the program PolyPhen-2 while it was predicted to be “not tolerated” at Seq Rep 0.45 in the Sorting Intolerant From Tolerant (SIFT) software program. Since the mutation resides in the triple-helical collagenous domain of type IV collagen α4 chain, its phenotype might be milder than that of the glycine mutation that appears in every three amino acids in the collagenous domain. There were also reports that TBMD progression might be associated with other modifier genes [16–20].

**Fig. 3:**
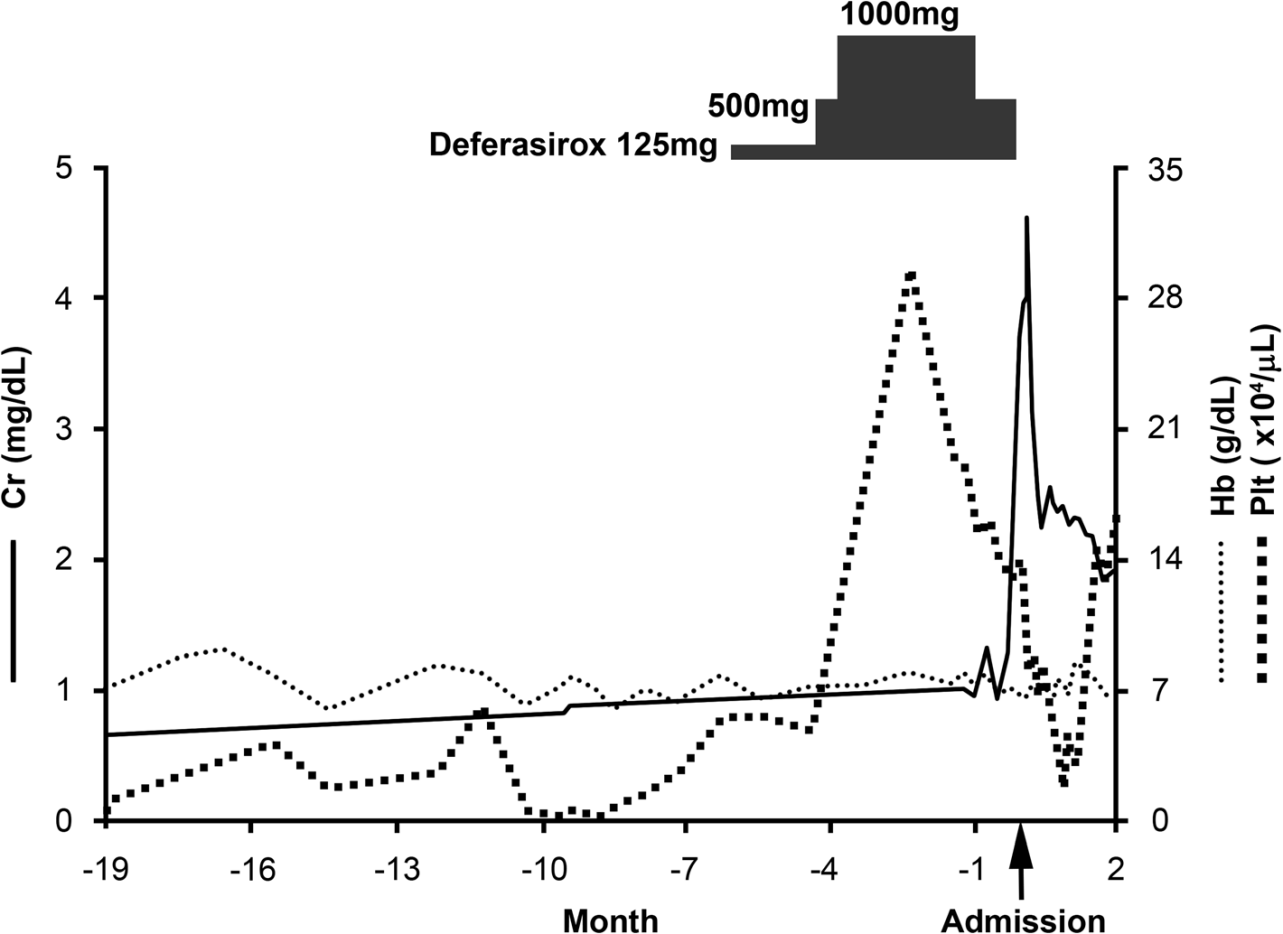
The clinical course. Admission (month 0). Cr; serum creatinine, Hb; hemoglobin, Plt; platelet

Gross hematuria is reported to occur in around 7% of adult TBMD patients [12]. However, loin pain hematuria syndrome was unlikely because there was no sign of lumbago. Although the patient had no coagulation disorders, thrombocytopenia due to MDS was noted (its nadir was 0.1 × 10^4^/μL, Fig. 3), which might have affected the predisposition to glomerular hemorrhaging under conditions of TBMD in the present case. The macroscopic hematuria in the present case did not occur when the thrombocytopenia was at its nadir, which might imply that an unknown mechanism—other than thrombocytopenia—was involved in the development of macroscopic hematuria. There was a report that described TBMD and acute kidney injury due to gross hematuria and tubular necrosis in a patient on warfarin therapy [21]. Another report described a case of TBMD and thrombocytopenia [22].

Since his renal function deteriorated rapidly and he had a fever, we suspected the patient had ANCA-associated vasculitis or post-infectious glomerulonephritis, and treatment with intravenous methylprednisolone was initiated. However, there was no evidence of crescent formation or mesangial proliferation in the glomeruli. This patient may have had some unknown infection rather than vasculitis because leukocytopenia due to MDS was observed.

Although Deferasirox is known to be nephrotoxic, gross hematuria is relatively rare. When we encounter a case of acute kidney injury with gross hematuria during treatment with Deferasirox, TBMD can be considered to be a cause of gross hematuria.

## Abbreviations

C3: Complement 3
Cr: Creatinine
GBM: Glomerular basement membrane
IgG: Immunoglobulin G
MDS: Myelodysplastic syndrome
MPO-ANCA: Myeloperoxidase-antineutrophil cytoplasmic antibody
RBC: Red blood cells
WF: Whole-field
